# The Effect and Potential Mechanism of Maternal Micronutrient Intake on Offspring Glucose Metabolism: An Emerging Field

**DOI:** 10.3389/fnut.2021.763809

**Published:** 2021-10-22

**Authors:** Yifan Wu, Qian Zhang, Xinhua Xiao

**Affiliations:** Key Laboratory of Endocrinology, Ministry of Health, Department of Endocrinology, Peking Union Medical College Hospital, Peking Union Medical College, Chinese Academy of Medical Sciences, Beijing, China

**Keywords:** DOHaD, diabetes mellitus, micronutrient, glucose metabolism, offspring, maternal, epigenetics

## Abstract

Diabetes has become the most common metabolic disease around the world. In addition to genetic and environmental factors in adulthood, the early life environment is critical to the progression of diabetes in adults, especially the environment during the fetal period; this concept is called “fetal programming.” Substantial evidence has illustrated the key role of early life macronutrient in programming metabolic diseases. Recently, the effect of maternal micronutrient intake on offspring glucose metabolism during later life has become an emerging field. This review focuses on updated human and animal evidence about the effect of maternal micronutrient status on offspring glucose metabolism and the underlying mechanism.

## Introduction

Diabetes is a serious metabolic disease and a major health concern in modern society. The prevalence of diabetes is high and increasing worldwide. According to the latest statistics, in 2019, the number of patients (20–79 years old) with diabetes worldwide reached 463 million, and it is expected to reach 700 million in 2045 (1). It is also a serious chronic disease that leads to various types of complications and an increase in disability and death, hence causing a huge problem of medical financial cost.

Type 2 diabetes mellitus (T2DM) accounts for 90% of diabetes cases (1). However, the causes of the disease are not fully understood. There is a modern perception that it results from a combination of multigene pre-disposition and environmental triggers. This perception was first put forward by James Neel in 1962 in his “thrifty genotype hypothesis.” He proposed the “thrifty genotype” as a trigger for an increased efficacy in utilizing food and fat deposition promotion under better nutrition status after starvation (2). In a study on the Dutch famine of 1994–1945, individuals born during famine have increased blood glucose levels, which was related to their birth weight (3). Moreover, subjects born during powerful tropical storms in Puerto Rico during the 1920s and 1930s are prone to hypertension, high blood cholesterol, and diabetes (4). Nevertheless, the hypothesis fails to explain why the “diabesity” pandemic is still spreading rapidly and widely in modern times with adequate availability of nutrients. In 1992, Barker et al. raised the “thrifty phenotype” hypothesis which summarized the relationship of the early life environment and the prevalence of metabolic diseases in epidemiological findings. The hypothesis was spread as the “Barker hypothesis” or “fetal programming hypothesis,” which suggests that unfavorable environmental factors during early life (intrauterine and postnatal) lead to a high incidence of metabolic diseases, such as hypertension, impaired glucose tolerance, insulin resistance, and T2DM (5, 6). This concept gradually evolved into the Developmental Origins of Health and Disease (DOHaD).

DOHaD clearly demonstrates the link between the adverse intrauterine environment and the risk for chronic metabolic consequences, including obesity, insulin resistance and cardiovascular disease, and such an effect may persist for generations through epigenetic mechanisms (7–9), including DNA methylation, histone modifications and non-coding RNAs (8). Epigenetic modifications are easily affected by environmental factors, such as tobacco smoking (10, 11), environmental toxins (12, 13), low-dose radiation (14), lack of physical activity (15–17), psychological stress (18), and circadian dysregulation (19, 20).

In addition, maternal nutrition status also affects epigenetic modifications, including protein deprivation and caloric excess. The initial evidence of the link is from severe famines. A 50% increase in T2DM development was seen among the Ukrainian population born during the Holodomor famine in 1932–1933 (21). In other studies on the Dutch Hunger Winter of 1944–1945, exposure to famine during gestation and childhood had life-long health effects, especially an increased incidence of impaired glucose tolerance (22, 23), and had transgenerational effects on offspring health later in life (24). Offspring DNA methylation changes were reported in those exposed to the Dutch Hungry in the periconceptional period (25, 26). Exposure to the Chinese famine from 1959 to 1961 in early life has increased the incidence of overweight and T2DM, which may be one of the explanations of obesity and T2DM epidemics in China (27). An increase in methylation levels in the insulin-like growth factor 2 (IGF2) and insulin receptor (INSR) genes was also observed in those exposed to severe famine in their early life (28, 29). In recent years, many studies have reported that maternal undernutrition, especially protein restriction, and overnutrition *in utero* resulted in the effects of fetal programming, which can be observed in animal models. Our group and other scientists' research also show that maternal protein restriction leads to pancreatic β-cell dysfunction in mouse offspring with the regulation of microRNAs (miRNAs), which may lead to offspring that are pre-disposed to insulin resistance and T2DM (30, 31). In addition, exposure of mouse pups to a high-fat diet in the fetal period leads to hyperglycemia, high serum leptin, and methylation modification disorder of the hepatic insulin receptor substrate 2 (*Irs2*) gene, the mitogen-activated protein kinase kinase 4 (*Map2k4*) gene, the hypothalamic proopiomelanocortin (*Pomc*) gene, and the melanocortin 4 receptor (*Mc4r*) gene (32, 33).

Most studies have focused on maternal macronutrient deprivation and excess during early life and its effects on later-life health. Maternal micronutrient malnutrition is also a threatening problem worldwide. Although micronutrients are needed in small amounts, they are essential to life activity and homeostasis. They are classified into minerals, trace elements and vitamins, which play an important role in biochemical reactions and function. Micronutrient deficiency usually leads to health problems (34, 35). Widespread global micronutrient deficiency poses a threat to people's health, especially pregnant women and their children's health, and results in poor growth, perinatal complications, and an increased risk of metabolic disorders later in life (36). Animal studies on the effects of maternal micronutrient deficiency or surplus on insulin resistance in offspring can be traced back to 2004, when Raghunath et al. found that maternal multimineral (Fe, Zn, Mg, and Ca) or multivitamin restriction led to early growth retardation, body composition alteration, and insulin resistance in offspring. Increased oxidative stress and/or decreased antioxidant defense in these offspring were also observed (37, 38). Since then, animal and clinical studies on the effects of single micronutrients, including minerals (Zn, Cr, Ca, Fe, Mg, Se, Zn) and vitamins (vitamins A, D, B12, and folic acid), on offspring metabolic status have been carried out successively. The possible mechanisms behind this phenomenon include increased oxidative stress and inflammation, insulin signaling pathways, eating reflex, etc. have been explored. In recent years, discussions on epigenetic mechanisms (such as DNA methylation and miRNAs) have gradually increased. In 2007, Sinclair et al. carried out a study in a sheep model, in which restricted dietary methyl donor (i.e., methionine and vitamin B12) provision up to day 6 following insemination in sheep altered DNA methylation and led to hypertensive and insulin-resistant offspring (39). This review summarizes the previous studies linking maternal micronutrient surplus and deficiency and its adverse impacts on glucose metabolism in offspring and the mechanisms involved in this process.

## Minerals

### Calcium

Ca is a key micronutrient in bone construction, blood clotting, and muscle contraction (40). The best natural sources of Ca are milk and dairy products. In addition, we can also obtain Ca from leafy green vegetables, nuts or fortified foods, such as flour and dairy alternatives (41). A study reported consistently low Ca intake in pregnant subjects across Asian, African, and Latin American countries due to the deficiency of dairy products consumption (intake between 200 and 500 mg/day) (42, 43). The recommended dietary allowance (RDA) of Ca set by the USA is 1,200 mg per day for men and non-pregnant women aged 31–50 (44). In pregnancy and lactation, women, particularly women with low dietary Ca intake, require an increase in their Ca intake (1,000–2,000 mg/day) to meet the Ca demands of both mothers and fetuses/infants (45, 46). Therefore, strategies of Ca supplement to achieve Ca recommendations should be targeted at those groups.

Ca restriction in pregnant women is significantly linked with abnormal offspring glucose metabolism. Several human studies have demonstrated that serum Ca levels are positively correlated with serum glucose levels and insulin secretion in subjects whose fast serum glucose <7.0 mmol/L (47) and changed oxidative stress is related with Ca dysregulation around T2DM patients (48). However, there is still a lack of robust clinical evidence on the association of maternal Ca intake and offspring glucose metabolism. Furthermore, relevant animal studies showed Ca deficiency is linked with the progress of offspring insulin resistance. Takaya et al. found that in rat models, a maternal Ca-deficient diet increased insulin resistance in the adulthood of offspring, especially in male offspring. This alteration may be involved in the influenced hepatic hydroxysteroid 11-beta dehydrogenase 1 (Hsd11b1) expression (49) and the altered osteocalcin, a bone formation biomarker, that acts directly on pancreatic β-cells and increases insulin secretion (50). The undercarboxylated osteocalcin (Glu-osteocalcin) was increased to 76.7 ± 48.4 ng/mL in female offspring fed on a maternal low Ca diet, compared with 19.6 ± 9.9 ng/mL in control group (50). Moreover, the influences of maternal Ca deficiency on insulin secretion were observed in the next three generations (51).

### Magnesium

Mg is an essential ion for protein production, muscle contraction, nerve transmission, and immune system health. Food sources of Mg usually include green leafy vegetables, nuts, legumes, and whole grains (52). Daily Mg intake at a dose of 310–360 mg/day (RDA) for girls/women aged 14 through 50 is recommended and the RDA for pregnant women for Mg increases to 350–400 mg/day. The range varies from adolescents to adults (53). The World Health Organization reported that the frequency of Mg deficiency is still problematic in general population in both developed and developing countries (54). A high percentage of pregnant women with low serum ionized Mg levels is found in the USA (55). Thus, pregnant women are still recommended to include good sources of Mg in their diets (56).

In glucose metabolism, Mg could improve insulin resistance. Widespread clinical evidence has proven that Mg intake deficiency and low serum Mg levels are related to T2DM, insulin resistance and metabolic syndrome (57). But the effect of maternal Mg restriction on offspring glucose metabolism currently lacks clinical evidence partly because the serum Mg levels cannot reflect the Mg stored in the body and the gold standard of Mg deficiency for pregnant women has not yet determined (56). Moreover, in rat models, Venu et al. found that maternal restriction of Mg in the diet resulted in an increase in body fat, induced insulin resistance, and impaired glucose tolerance in male pups and that rehabilitation with Mg supplementation can partially correct body composition and low-birth weight by 6 months of age (58). However, in another study, rehabilitation of Mg in the diet of the male pups of rats showed no correction of body composition at 18 months (59). The inconsistency between the two studies is likely to result from the different ages of the offspring.

Abnormal Mg balance is associated with the pathogenesis of T2DM. However, the plausible molecular mechanisms involved are still not fully understood. In carbohydrate metabolism, Mg serves as a critical cofactor of many enzymes (60). First, Mg deficiency impairs glucose intake in the endothelia of target tissues, including liver and adipose tissue, by inhibiting glucose transporter 4 (GLUT4) expression. In addition, low serum Mg affects insulin secretion and signaling because Mg acts as an insulin sensor to induce insulin receptor phosphorylation and regulate reporter tyrosine kinase activity. Furthermore, Mg deficiency is also associated with an impaired antioxidant system of endothelial cells, oxidative stress, and lipid metabolism disorder (61, 62). In an animal study on maternal Mg deficiency, the expression of fatty acid synthase (FASN) and fatty acyl transport protein 1 (FATP 1) in liver and adipose tissue was increased in offspring at 18 months of age (59). Additionally, chronic Mg intake deficiency in WNIN female rats not only increased serum corticosterone, leptin, and proinflammatory cytokine levels but also led to lipid metabolism disorder and insulin resistance in their offspring (63).

### Chromium

Cr is an essential trace element that is important in carbohydrate metabolism. The adequate intake (AI) is 25 and 30 mcg/day for non-pregnant women and pregnant women, respectively (64). The data on Cr intakes from dietary supplements are very limited; thus, the frequency and degree of Cr restriction remain unclear (65). It is found as a cofactor in insulin signaling. Diabetic and prediabetic patients usually have low serum Cr concentrations (66).

The potential mechanisms of the link between Cr and glucose metabolism are as follows. The Cr-nicotinic acid complex has been proven to be a glucose tolerance factor (GTF) that potentiates the action of insulin *in vitro* and *in vivo* (67, 68). Cr also plays a potential anti-inflammatory role in glucose metabolism (69). Cr restriction leads to glucose and lipid metabolism disorders (70). Cr supplementation intake moderates fasting blood glucose levels in diabetic patients (71, 72) and diabetic rat models (73). The involved mechanism is mainly insulin signaling moderation, such as insulin binding, insulin receptor number, insulin internalization, pancreatic β cell sensitivity and insulin receptor enzymes (74).

Additionally, Cr intake in pregnant women can also affect glucose metabolism in offspring later in life (65). In a clinical study (*n* = 76), Cr levels in hair and blood samples of infants of diabetic mothers were significantly lower than those of infants from healthy mothers (age ranged 30–40) (75). Several animal studies have reached the same conclusion that maternal Cr restriction contributes to pre-disposal to diabetes and obesity in offspring, and the underlying mechanisms are complex. A study in 2010 reported that maternal Cr restriction increased body fat of the offspring at 12 months of age in both genders and was associated with impaired glucose uptake by muscle in WNIN rat offspring (76). Increased oxidative stress is also an underlying mechanism (77). Additionally, maternal Cr restriction inhibits hepatic insulin signaling and activates Wnt signaling (78). Our group data also found miRNA dysfunction and alteration of the methylation status of hepatic genes in animal models (79, 80).

### Iron

Fe is an essential mineral functioning as a coenzyme in a variety of physiological processes, including oxygen transportation, red blood cell growth, energy metabolism and fat metabolism, immune response, and DNA synthesis and repair (81–83). Fe restriction leads to adverse outcomes during pregnancy. It is also a worldwide health problem, especially for infants, nursing mothers, and women of reproductive age (84, 85). The physiologic demand for Fe considerably increases during pregnancy compared with the average amount of Fe intake, changing from 0.8 mg to at most 7.5 mg absorbed Fe per day (86), increasing the risk of anemia in pregnant women. The adverse pregnancy outcomes of Fe deficiency include intrauterine growth retardation, pre-maturity, fetoplacental miss ratio, and higher risk for peripartum blood transfusion (87).

However, excess Fe, stored in the liver, influences glucose metabolism and leads to insulin resistance and T2DM (88). Plasma Fe overload is positively correlated with the risk of insulin resistance and T2DM, although it does not typically feature T2DM (89). Pregnant women with increased Fe stores are at higher risk of developing gestational diabetes mellitus (90). Transferrin saturation was 3–4-fold higher in patients with T2DM (91). Elevated Fe stores may be induced through multiple pathways, such as pancreatic β cell oxidative damage, hepatic insulin extraction impairment, and hepatic glucose production suppression (92, 93). Psyrogiannis et al. indicated that offspring from T2DM parents have relative Fe “overload,” which may be a major reason why insulin resistance worsens in offspring (94, 95). Furthermore, in rodent models, Zein et al. found that excessive Fe intake in pregnant mice on a high-fructose diet increased blood sugar in male offspring mice and body weight in female offspring mice, which appeared to be associated with decreased activities of the antioxidant enzyme glutathione peroxidase (GPx) (96). Thus, a high Fe intake increases the risk of glucose metabolism disorder and oxidative damage in newborns, indicating that we should take a more cautious attitude toward Fe supplementation during gestation. According to the Food and Nutrition Board (FNB), daily Fe intake range is 27–45 mg [RDA and upper intake level (UL)] for pregnant women in order to reduce the risk of both Fe restriction and excess due to the aforementioned reasons (64).

### Selenium

Se, another trace element, binds to proteins to make Se-dependent glutathione peroxidases and other selenoprotein complexes to defend against oxidative stress (97). Se intakes and serum concentrations vary somewhat by geographical region because the amounts of Se in soil and in local foods differ from each other regions (98). Se deficiency diseases, Keshan disease, have been exclusively reported in low-Se rural areas in China (99). Se has a dual effect on glucose metabolism: Se serves as an antioxidant to prevent the progression of T2DM and its complications, while excessively high serum Se levels are related to a high prevalence of diabetes (100–102). Administration of Se (0.2 μmol/μL) in drinking water in non-obese diabetic mice for 3 weeks reduced fasting blood glucose and moderate lipid metabolism (103). In a follow-up study over two decades, individuals with high serum Se levels had a 24% reduction in diabetes risk, compared with those with low serum Se levels (104). In one cross-sectional analysis of diabetic patients and healthy people, GPx activity declined significantly, and a slight decrease was observed in the concentration of total Se, indicating the association of Se levels and the progression of diabetes (105). Therefore, Se supplementation is an efficient way to prevent glucose metabolism disorder because of the connection of its counteractive oxidative properties and the onset of metabolic diseases, such as T2DM. Conversely, a cross-sectional analysis of American adults discovered that high serum Se is positively correlated with the prevalence of diabetes (101). Additionally, a meta-analysis published in 2018 comes to the same conclusion (106). The potential explanation behind this difference is that serum Se reflects only the amount of Se intake and not the activity of selenoprotein, the form participating in the biologic function of Se. More studies are needed to explain the association and mechanism of excessive Se intake and glucose metabolism disorder.

During pregnancy, serum Se levels decrease significantly in the first few months of life. This reduction in the pregnant mother initiates oxidative stress in the fetus (107). Adequate ingestion of Se is basic nutrition, as its deficiency results in pregnancy-related complications, metabolic disorders, and other disorders in offspring (108, 109). Melo et al. found that in rats, maternal Se increases insulin secretion and glucose tolerance at 80 days of age (110). However, Zeng et al. found that excessive Se in the mother's diet led to insulin resistance in offspring at 112 days of age (111). The different conclusion between the two studies may arise from the different dosages in the maternal diet, as both the low-Se and high-Se diets during pregnancy can induce insulin resistance with different mechanisms (112).

The recommended Se intake by the FNB is stipulated at levels of 60 mg/day (RDA), changing from 55 mg/day for non-pregnant women to assure the optimal concentration of GPx in blood serum; the UL of Se intake is set at 400 mcg per day (113). A report published by the German, Austrian and Swiss nutrition societies suggested that as persons from Se-deficient regions (China) can achieve selenoprotein P (SelP) saturation by taking a daily intake of 49 mcg of Se, they recommend a daily Se intake of 70 mcg for men and 60 mcg for women, whether pregnant or not (114). Moreover, People living in areas with low Se soil concentration are recommended to increase Se intake (114).

### Zinc

Zn directly affects the physiology and action of insulin (115), the regulation of cytokine expression, the suppression of inflammation, and the activation of antioxidant enzymes (116). Zn is important in the functions of epigenetic enzymes; thus, lack of Zn may disrupt the bioactivities through epigenetics mechanisms (117). Zn can also increase glucose transport into cells and potentiate insulin-induced glucose transport to moderate glucose and lipid metabolism (118).

Inadequate Zn intake is fairly common globally (119), especially in some select population subgroups, including lower socioeconomic groups, vegetarians, the elderly, and pregnant and lactating women (64, 120, 121). Pregnant women require an increased Zn intake, changing from the recommended amount of 18–27 mg/day (RDA), thus the risk of Zn deficiency around pregnant women is increased (64). Low serum Zn levels in pregnant women are related to several pregnancy complications (122). Adequate Zn supplementation during pregnancy reduces the risk of pre-term birth (123, 124). Zn deficiency is likely a reflection of poor nutrition, thus the best method to improve Zn deficiency is overall maternal nutrition supplementation (125). A randomized clinical study in Peru in 2017 did not reveal any evidence of the effect of maternal Zn restriction on insulin resistance (126). In contrast, many rodent studies have shown the link between Zn deficiency in pregnant rats and insulin resistance in their offspring. The first animal study was conducted by Rosario et al., in which they found that maternal Zn deficiency played a key role in the metabolic status of the offspring (127). In addition, decreased expression of placental hydroxysteroid 11-beta dehydrogenase 2 (HSD11B2) was observed in pregnant women having a Zn restriction diet, which increased fetal exposure to maternal cortisol and may lead to insulin resistance and hypertension in the offspring (128). In addition, Padmavathi et al. suggested that maternal Zn restriction not only induces insulin resistance, but also alters body composition and adipose profile (129). The alteration is associated with serum leptin levels and sex-specific changes in the insulin signaling pathway. Additionally, the effect of maternal Zn restriction is related to the nutrient intake of the offspring, as offspring fed an excessive nutrient diet had higher weight and insulin-like growth factor-1 (IGF1) levels (130, 131). Therefore, Zn restriction in rats in early life impairs downstream insulin signaling with sex differences, and Zn supplementation at weaning reverses the adverse effect (132).

## Vitamins

### Vitamin A

Vitamin A is the general term for retinol and its natural metabolites, including retinal and retinoic acid. About 10–20% of pregnant women develop vitamin A deficiency in developing countries, and subclinical vitamin A deficiency affects more individuals, particularly in Africa and Asia (36). Carotenoids such as B-carotene can be converted into retinol in the human body. These substances are collectively called carotenoids (133). In the past, the physiological functions of vitamin A and carotenoids were widely studied. They play an important role in reproduction, embryogenesis, vision, growth, cell differentiation and proliferation, maintenance of epithelial cell integrity and immune function (134–136). Studies on the link between vitamin A and metabolic diseases, such as obesity and T2DM, are receiving increasing attention (137, 138).

In a Denmark birth cohort study, high prenatal vitamin A exposure was related to a low risk of developing T2DM (139). Retinol binding protein 4 (RBP4) is a novel type of adipokine that affects vitamin A delivery to tissues. In 2005, Kahn et al. found high serum RBP4 levels in insulin-resistant mice and obese and T2DM individuals, which may participate in the pathogenesis of T2DM (140, 141). In addition, vitamin A is important in the development of the pancreas, especially the differentiation and growth of pancreatic β-cells (138, 142, 143). Impaired fetal pancreas function was seen in rodent models of vitamin A deficiency (144, 145). However, Guo et al. found that excessive supplementation of β-carotene during pregnancy disturbed the lipid metabolism of the offspring and induced impaired glucose tolerance in rat models (146). Thus, the proper intake of vitamin A and carotenoids in pregnancy is needed to avoid both maternal excess and deficiency intake. The recommended amount of vitamin A intake was given as retinol activity equivalents (RAE). The range of vitamin A intake is 700–3,000 mcg RAE/day for non-pregnant women, 770–3,000 mcg RAE/day for pregnant women, and 1,300–3,000 mcg RAE/day for lactation women (64).

### Vitamin D

Vitamin D is a lipid-soluble steroid and an anti-inflammatory factor. The major physiological action of vitamin D is bone health. Its main resources include dietary intake and skin synthesis through sun exposure. After two hydrogenation conversions, vitamin D transforms into its active form, 1,25-dihydroxyvitamin (OH)_2_D, and then binds to vitamin D receptor (VDR) in the nuclear receptor and affects gene transcription, including calcium binding protein (CaBP), epithelial calcium channel (ECaC), 25(OH)D 24-hydroxylase (24-OHase), receptor activator nuclear factor-κB ligand (RANKL), alkaline phosphatase (alk PASE), prostate-specific antigen (PSA), and parathyroid hormone (PTH) (147, 148). As vitamin D can be obtained by receiving regular exposure to sunlight, it is recommended that pregnant women do not need extra vitamin D supplementation; thus, women, whether pregnant or not, are recommended to intake 5 mcg per day (53). Vitamin D enables the intestine to absorb more Ca and phosphorus and moderates the renal tubules reabsorbing Ca. The existence of VDR in the heart, liver, blood vessels, and central nervous system and other organs means that vitamin D also functions in these tissues (149). Vitamin D deficiency is associated with the risk of both type 1 diabetes mellitus and T2DM (148, 150, 151). In a cross-sectional study of 808 non-diabetic participants of the Framingham Offspring Study, plasma 25(OH)D concentrations were inversely associated with markers of the insulin resistant phenotype (152). The mechanism of the effects of vitamin D on glucose metabolism includes regulating insulin production and secretion, enhancing insulin sensitivity, and reducing inflammation (147, 153). In terms of epigenetics, vitamin D participates in the increased expression of DNA demethylases that inhibit hypermethylation of multiple gene promoter regions of many diabetes-related genes (154). High vitamin D intake during pregnancy results in an increase in maternal and infant 25(OH)D concentrations and engages in maternal glucose metabolism and fetal growth (155).

In recent decades, many studies have shown that vitamin D deficiency during pregnancy may potentially affect chronic disease susceptibility of fetuses later in life (156). Many clinical studies have proven that maternal vitamin D deficiency is linked with increased insulin resistance in offspring in early childhood (157–159). Rehabilitation of maternal and offspring vitamin D reduces fasting glucose levels, as shown in rat models (160). The underlying mechanism may be involved in the epigenetic alterations of special genes. In animal models, vitamin D deficiency during pregnancy contributed to insulin resistance and impaired glucose tolerance in male offspring, associated with increased DNA methylation of the nuclear factor κB inhibitor α (*I*κ*b*α) gene, which decreased luciferase activity (161).

### Vitamin B12 and Folate

Vitamin B12 is a micronutrient essential for one-carbon metabolism. Lack of vitamin B12 may result in decreased RBC production, dysfunction of the nervous system, and metabolic disorders. During pregnancy, vitamin B12 is essential for fetal growth and development. The metabolic demands of fetuses for vitamin B12 are increasing during pregnancy (162). Vitamin B12 and folate deficiency mainly result from poor dietary intake, increased demand for maternal biological activity and fetal growth, and the increased loss of folate (163). Recommended vitamin B12 intake for pregnant women is 2.6 mcg per day, rising from 2.4 mcg per day for non-pregnant female adults (164). The FNB introduced dietary folate equivalents (DFEs) when calculating different absorption of folate. Individuals who are pregnant should consume 600 mcg DFE daily, increasing from 400 mcg DEF per day for non-pregnant women (164). However, maternal vitamin B12 deficiency has become increasingly common (165).

Vitamin B12 and folate deficiency in pregnant women are more likely to cause insulin resistance. In a clinical study in Pune, low maternal vitamin B12 and high folate status may increase the prevalence of obesity and T2DM in India (166). Other studies, one from Nepal in 2011 and one from India in 2014, obtained consistent findings that maternal vitamin B 12 deficiency is related to a high risk of insulin resistance in school-aged offspring (167, 168), but folate supplementation showed no significant improvement in insulin resistance among offspring at 6–8 years of age (167). Additionally, in a study in the UK, such changes were not observed in the cord blood of neonatal Caucasian patients (169). These findings indicated that the effects of maternal vitamin B12 restriction on offspring may not be observed immediately in early life. Additionally, in a systematic review including 46 articles in India, low maternal vitamin B12 status was associated with a high risk of adverse health outcomes (high adiposity, insulin resistance, and low offspring B12) in offspring (170).

Apart from many human studies, many animal models also provide substantial evidence and reveal the potential mechanisms of the relationship between maternal vitamin B12 and folate status and glucose metabolism in offspring. The first animal study is conducted by Sinclair et al. They found an association of folate reduction in maternal diet and alterations to DNA methylation and increased insulin resistance in offspring (39). The effect of vitamin B12 and folate is related to the offspring diet. Several animal studies found that in female offspring, increased body fat percentage, fasting hyperglycemia, and insulin resistance in later life associated with a maternal high folic acid and low vitamin B12 intake; though dependent on offspring diet intake (171, 172). Furthermore, vitamin B12 supplementation may partially reverse the adverse effect, but the timing is critical. In another study, rehabilitation at weaning did not show any improvement in the onset of adverse outcomes, but rehabilitation at parturition did (173). In another rodent study, altered DNA methylation in metabolic-related genes in the offspring of maternal vitamin B12 restriction was reported, and rehabilitation at conception and parturition partly reversed the methylation status (174).

## Conclusion and Future Perspective

**Figure 1:**
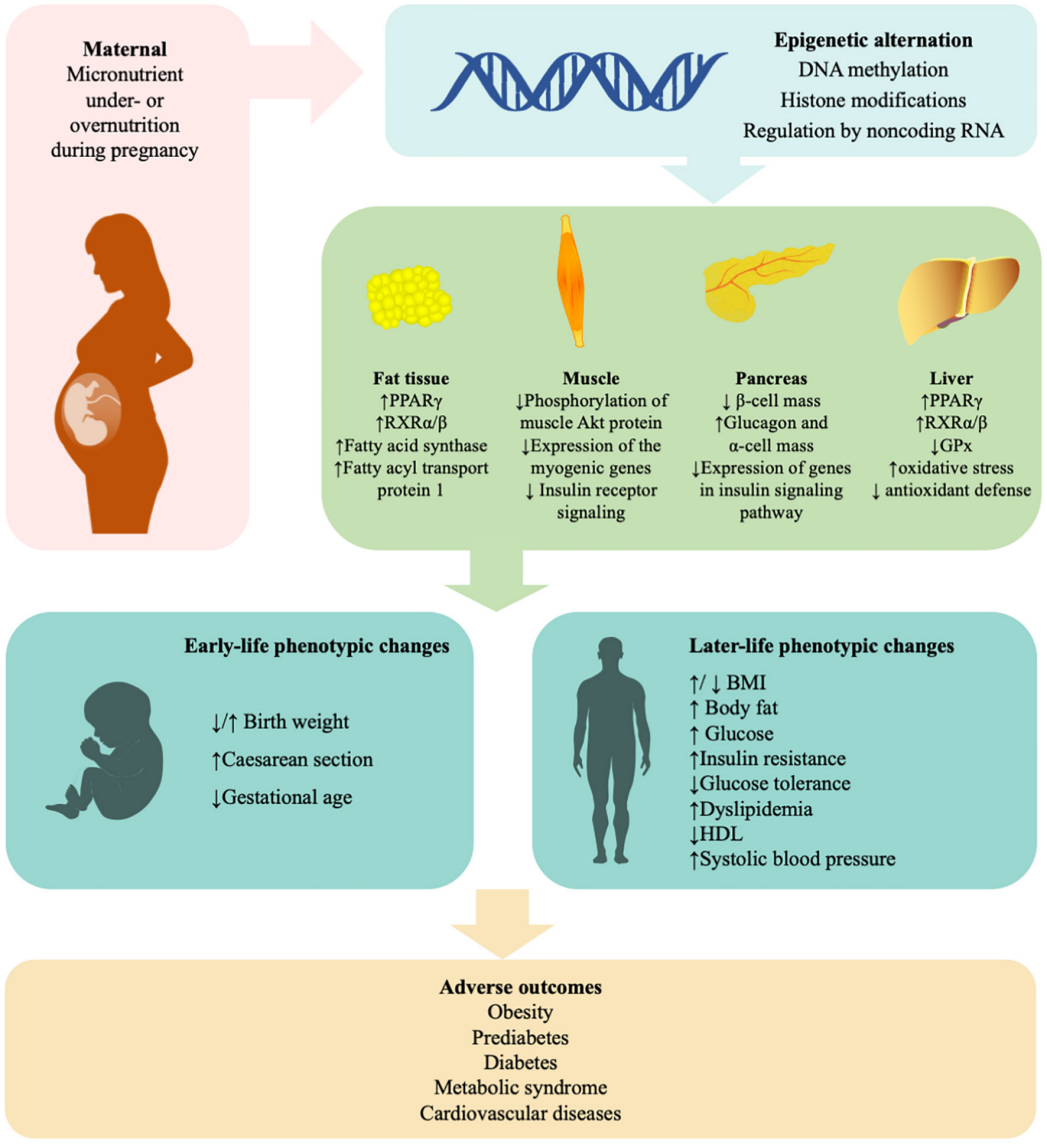
Mechanisms in the effects of maternal micronutrient on glucose metabolism of offspring. “Fetal programming” suggests that maternal micronutrient is linked with the susceptibility of insulin resistance, glucose intolerance, obesity, and type 2 diabetes in offspring. Epigenetic mechanisms, including DNA methylation, histone modifications, and microRNA, play a critical role in the process. Supplement of minerals and vitamins under medical guides will be beneficial to subsequent generations. BMI, body mass index; GPx, glutathione peroxidase; HDL, high-density lipoprotein; PPARγ, peroxisome proliferator-activated receptor gamma; RXR, retinoid X receptors. ↑, increased; ↓, decreased.

It is obvious that both maternal excess and deficiency of micronutrients affect glucose metabolism and induce insulin resistance in offspring later in life. Micronutrient deficiency largely results from poverty. Other possible causes of undernutrition and overnutrition include sociocultural factors, economic factors, inadequate care and feeding practices, improper dietary intake and diseases (36). The mechanisms are summarized in Figure 1. Recently, robust evidence from human and animal studies has convincingly shown that improper micronutrient intake in pregnant women induces epigenetic alterations in the placentae and fetuses that can alter the expression of genes in offspring, influence fetal growth and alter glucose metabolism in adulthood. These epigenetic factors are heritable for several generations. A list of human studies is summarized in Table 1 and a list of animal studies is in Table 2. Therefore, it should be seen as equally significant compared with genetic factors in the discussion of the risk of T2DM. However, it is still unclear how much micronutrient intake is proper in different gestational stages to avoid adverse effects on glucose metabolism and how many generations it induces epigenetic alterations. In addition to micronutrient intake, other environmental factors, including parental obesity/diabetes, macronutrients, toxins, stress, and physical activities, should also be taken into consideration because they also result in the inherited traits and contribute to diabetic offspring later in life.

**Table 1:** Charting the maternal micronutrients status and the effect on glucose metabolism in offspring.

**Study design (reference)**	**Participants**	**Age of offspring**	**Maternal status**	**Effect on offspring**
RCT (126)	Peru *n* = 159	4.5 years	60 mg Fe + 250 μg folic acid + 25 mg of Zn (control) 60 mg Fe + 250 μg folic acid	↑Abdominal obesity, ↓HDL, and ↓HOMA-IR (no statistical significance)
Retrospective analysis (139)	Denmark *n* = 193,803	36 years	Pregnant mothers exposed to extra vitamin A	↓Risk of developing T2DM
Prospective observational cohort study (157)	Amsterdam *n* = 1,882	5–6 years	Pre-pregnancy BMI ≥ 25.0 kg/m^2^ and maternal low 25 OHD	↓C-peptide and insulin resistance
Prospective study (159)	India *n* = 568	9.5 years	Vitamin D deficiency (<50 nmol/l)	↓Arm-muscle area; ↑fasting insulin resistance
Prospective study (166)	Pune *n* = 653	6 years	Low vitamin B12 (<150 pmol/l)	↓BMI and weight; ↑adiposity and insulin resistance
RCT (167)	Nepal *n* = 1,132	6–8 years	Vitamin B12 deficiency (<148 pmol/l)	↑HOMA-IR; association not dependent on folic acid
Observation cohort study (168)	India *n* = 654	9.5 and 13.5 years	High maternal folate concentrations	↑HOMA-IR
Cross-sectional study (169)	Caucasian in the UK *n* = 182	Newborns	Low serum vitamin B12 (<191 ng/l) and folate (<4.6 μg/l)	↑HOMA-IR, triglycerides, homocysteine; ↓HDL-cholesterol

*BMI, body mass index; HDL, high-density lipoproteins; HOMA-IR, homeostasis model assessment 2-insulin resistance; N/A, not applicable; RCT, randomized controlled trial; ↑, increased; ↓, decreased*.

**Table 2:** Summary of animal studies investigating the effects of maternal micronutrients on glucose metabolism in offspring.

**Model (Reference)**	**Maternal status**	**Age of offspring**	**Phenotypic observations**	**Tissues**	**Epigenetic alteration/expression change**
Wistar rats (50)	Ca-deficient diet	180 days	Male: ↑insulin, glucose, and HOMA-IR; ↑adiponectin; ↑leptin. Female: ↑Glu-OC and Gla-OC	Serum	N/A
Wistar rats (51)	Ca-deficient diet	180 days	F1, F2, and F3: ↓HOMA-β% F1 males: ↑insulin and HOMA-IR F3 males:↓insulin and HOMA-IR Females: no differences in insulin and HOMA-IR	Serum	N/A
Wistar rats (49)	Ca-deficient diet	200 days	Male: ↑body weight; ↑insulin, glucose, and HOMA-IR; ↑adiponectin Female: ↑body weight, ↑insulin	Liver	Male: ↓*Hsd11b1* mRNA levels Female: ↑*Hsd11b1* mRNA levels
WNIN rats (58)	Mg-restricted diet	180 days	Male: ↓weight; ↑body fat, ↓lean body and fat-free mass; ↑insulin and HOMA-IR; ↑insulin resistance; rehabilitation at parturition and weaning partially corrected body composition Females: not mentioned	Serum	N/A
WNIN rats (59)	Mg-restricted diet	18 months	Male: ↑body fat; ↓LBS and FFS; ↓glucose-stimulated insulin secretion and basal glucose uptake by the diaphragm; ↓plasma leptin; ↑TNF-α and corrected by rehabilitation from parturition but not from weaning Females: not mentioned	Liver and adipose tissue	Male: ↑FASN and FATP 1 levels
WNIN rats (63)	Mg-Restricted diet	Gestational 15 days	↑Corticosterone and proinflammatory cytokines	Placentae and fetuses	↑*Hsd11b1* mRNA levels; ↓*Hsd11b2* mRNA levels
WNIN rats (77)	Cr-restricted diet	15 months	Both genders: ↑Fasting plasma glucose, fasting insulin; ↑HOMA-IR	Liver and pancreatic	Male: ↑expression of genes involved in insulin secretion Both genders: ↓antioxidant enzyme activities
WNIN rats (76)	Cr-restricted diet	N/A	Unclear gender: ↓LBM% and FFM%; ↑basal glucose uptake by muscle; No change in insulin sensitivity	Muscle	↓Expression of myogenic genes: *MyoD, Myf5*, and *MyoG*; ↓expression of the muscle atrophic genes: *Atrogin* and *MuRF1*
C57BL mice (79)	Low-Cr diet	16 weeks	Male: ↑fasting serum glucose and serum insulin; ↑insulin resistance and AUC of blood glucose Female: not mentioned	Liver	Male: MicroRNA alteration in insulin signaling pathway Female: not mentioned
C57BL mice (80)	Low-Cr diet	29 weeks	Male: ↑body weight, blood glucose, and serum insulin levels; Impaired glucose tolerance and insulin resistance Female: not mentioned	Liver	Male: ↑DNA methylation associated with insulin signaling pathway; ↓expression of genes in insulin signaling pathway Female: not mentioned
C57BL mice (78)	Low-Cr diet	32 weeks	Male: ↑Body weight, fasting blood glucose, AUC of blood glucose, fasting insulin, and HOMA-IR Female: not mentioned	Liver	Male: Changed expression of genes in insulin signaling pathway and Wnt signaling pathway Female: not mentioned
Wistar rats (96)	Fructose-rich diet (F) Fructose iron-enriched diet (FI)	At delivery	F diet: ↓glycemia FI in male offspring: ↑glycemia; FI in female offspring: ↑body weight	Liver and brain	FI in male offspring: ↓brain GPx activity; ↓liver GSH concentration; ↑activity of GST; FI in female offspring: ↓liver GPx activity
Wistar rats (110)	Sodium selenite treatments	80 days	Male: ↑milk intake and the visceral white adipose tissue; ↓insulin resistance; ↑glucose-stimulated insulin secretion; ↓triglycerides Female: not mentioned	Liver	Male: ↑*pgc-1*α and *nrf 1* mrna levels Female: not mentioned
Wistar rats (111)	Basal diet + Se	112 days	↑Insulin, insulin resistance, and glucose intolerance	Liver and muscle	↓mRNA and protein levels of insulin signal proteins;
				Pancreas, liver, and erythrocytes	↑GPx1 mRNA or GPx1 activity
				Liver	↓*Selh, Selp*, and *Selw* mRNA levels; ↑*Sels* mRNA levels
Wistar rats (112)	Se supplemented (ss) Se deficient (sd)	21 days	Ss: ↑milk intake and BMI; ↑Se in serum and liver; ↑triglycerides; ↑insulin and HOMA-IR sd: ↑milk intake, ↓body weight; ↓Se in liver, pancreas, and blood; ↑triglycerides and cholesterol; ↑urea and creatinine; ↓insulin and HOMA-IR	Liver	Ss: ↓irs-1 levels, ↑antioxidant enzymes; ↑GPx1 and Selp levels; ↓the ratio pampk/tAMPK sd:↓irs-1 levels, ↓antioxidant enzymes ratio; ↓GPx1 and Selp levels; ↓tampk levels, ↑pampk levels
Wistar/nin rats (129)	Zn-restricted diet	6 months	↓Body weight; ↑body fat and ↓LBS and FFS; rehabilitation only corrected the body weights of male but not female offspring. Both genders: ↓fasting plasma insulin levels; not corrected; ↓glucose tolerance and insulin response; not corrected	Serum	N/A
Sprague dawley rats (131)	Znd (maternal Zn-restricted diet)-an (adequate nutrition)/in (inadequate nutrition)/en (excess nutrition)	1.5 weeks and 3 weeks	Znd-in: ↓body weight; Znd-en: ↑body weight;	Serum and liver	N/A
		3 weeks	Znd-IN: ↓serum Zn; Znd-en and Znd-an: ↑serum C-peptide; Znd-en: ↑serum insulin; ↑leptin; Znd-an: ↑serum igf1		
		10 weeks	Znd-en and Znd-an: ↑insulin resistance;		
		15 weeks	Znd-en and Znd-an of male offspring: ↑serum C-peptide; ↑insulin; ↑leptin; female offspring: no differences Znd-en and Znd-an of female offspring: ↓insulin receptor phosphorylation in liver Znd-en of male offspring: ↓insulin receptor phosphorylation in liver		
Sprague dawley rats (130)	Zn-deficient diet	10 days and 20 days	↑Body weight;	Serum and muscle	N/A
		3 weeks	↑Blood glucose and serum igf1 concentration		
		5 and 10 weeks	↓Insulin sensitivity;		
		15 weeks	Male: ↑leptin; Female: ↓phosphorylation of muscle akt protein		
Wistar rats (132)	Low-Zn diet	81 days	Male: ↑systolic blood pressure, hyperglycemia, hypertriglyceridemia, insulin resistance and serum igf1 concentrations; ↑lipid peroxidation and catalase activity; rehabilitation reversed the effects female: less sensitive to the metabolic effects of zinc restriction.	Serum and liver	N/A
Sprague Dawley rats (144)	A diet containing retinol as retinyl palmitate	65 days	↓β-cell area and number ↓The percentage of newly replicated β-cells ↓Lower plasma insulin level and ↑serum glucose	Serum and pancreas	N/A
Rats (146)	B-carotene in pregnant mice	125 days	Female: ↑visceral fat; ↓glucose intolerance; ↑serum leptin, resistin, and IL-6 and ↓adiponectin; ↑pancreatic β-cell mass, glucagon, and α-cell mass	Liver, muscle, and adipose tissue	Female: ↑pparγ and rxr; ↓insulin receptor signaling in muscle and insulin gene; ↑*Gcg* gene
Sprague-Dawley rats (161)	A vitamin-d-free diet	16 weeks	↑Fasting insulin and HOMA-IR; ↓glucose tolerance and ↑insulin resistance; ↑serum and liver iL-1β, IL-6, IL-8, and tnf-α concentrations	Serum and liver	↓Expression of hepatic *iκbα* mRNA and protein
C57BL/6J rats (160)	A high vitamin d3 diet	7 months	↓Fasting glucose and serum lps concentrations; ↓epididymal fat pad relative weight	Serum	N/A
Wistar rats (174)	Vitamin B12-restricted diet	12 months	↓Birth weight, ↑adiposity, insulin resistance, and triglyceride levels; rehabilitation partially reversed DNA methylation	Liver	↑DNA methylation involved in fatty acid metabolism and mitochondrial transport or metabolism
Wistar rats (171)	Supplemental folic acid with no b12	35 weeks	Control diet-fed offspring: ↑fasting hyperglycemia, glucose intolerance, ↓β-cell mass western diet-fed offspring: ↓fasting blood glucose and plasma insulin concentrations, ↓weight	Liver	Control diet-fed offspring: ↑islet *Hnf1a* and *Nr1h3* mrna levels
C57BL/6J mice (172)	A folic acid-supplemented diet	23 weeks	↓Visceral and subcutaneous adipose tissue; ↓Serum total adiponectin and vitamin b12 concentrations	Aorta	↓NOX2 levels
C57BL/6J mice (173)	Vitamin b12-restricted diet	12 and 36 weeks	↑Body fat percentage, visceral adiposity, dyslipidemia, fasting hyperglycemia, and insulin resistance rehabilitation is beneficial	Serum	N/A
Scottish blackface sheep (39)	Cobalt and sulfur, restricted diet	12 and 22 months	↑Weight; ↑insulin resistance, and ↑immune responses to antigenic challenge	Liver	Altered methylation status in fetal liver at gestational day 90
		23 months	Males: ↑blood pressure		

*Akt protein, threonine kinase; AMPK, adenosine monophosphate-dependent kinase; Atrogin, MAFbx; AUC, area under the curve; BMI, body mass index; FASN, fatty acid synthase; FATP 1, fatty acyl transport protein 1; FFS, fat free mass; Gcg, glucagon; Gla-OC, carboxylated osteocalcin; Glu-OC, undercarboxylated OC; GPx, glutathione peroxidase; GSH, glutathione; GST, glutathione-S-transferase; Hnf1a, hepatocyte nuclear factor 1 homeobox α; Hsd11b1, hydroxysteroid 11-beta dehydrogenase 1; Hsd11b2, hydroxysteroid 11-beta dehydrogenase 2; HOMA-IR, homeostasis model assessment-insulin resistance; HOMA-β%, homeostasis model assessment of β-cell function; iκbα, nuclear factor κB inhibitor α; IRS-1, insulin receptor substrate 1; IGF1, insulin-like growth factor-1; LBS, lean body mass; MuRF 1, muscle RING finger; Myf5, myogenic factor 5; MyoD, myogenic differentiation; MyoG, myogenin; N/A, not applicable; NOX2, NADPH oxidase 2; Nrf 1, nuclear respiratory factor-1; Nr1h3, nuclear receptor subfamily 1 group h member 3; Pgc-1α, peroxisome proliferator-activated receptor-gamma coactivator 1-α; PPARγ, peroxisome proliferator-activated receptor gamma; pAMPK, phosphorylated AMPK; RXR, retinoid X receptors; Selh, selenoprotein H; SelP, selenoprotein P; Sels, selenoprotein S; Selw, selenoprotein W; tAMPK, Total AMPK; TNF-α, tumor necrosis factor-α; ↑, increased; ↓, decreased*.

However, some limitations should be noted. Due to the lack of clinical studies, we largely focus on rodent experiments. Currently, clinical studies on micronutrients are relevantly difficult to conduct because of the difficulties in excluding possible confounding factors, controlling daily micronutrient intake, measuring dietary micronutrients intake at the microgram level and other methodologic challenges. The small numbers of participants involved in studies on certain micronutrient and limited follow-up are also disadvantages for further analysis. Therefore, the discussion on clinical studies is limited and lacks quantitative, detailed analysis.

In the process of our learning how epigenetic factors participate in the development of T2DM and its rapid expansion, there remains much more to be explored. How are DNA methylation, histone codes or non-coding RNAs involved in the process? When is the key period to develop epigenetic alterations? When should interventions be conducted during gestation to target specific epigenetic markers? Moreover, considering the period prior to conception or a certain time when pregnancy is not yet known, but nutrient requirements are essential, more questions require answers. The questions are given below. What is the relationship between the micronutrient status in these stages and offspring glucose metabolism later in life? How does micronutrient status affect offspring glucose metabolism during these periods? And what strategies can be adopted to optimize micronutrient status at that time? Finally, today, there are many limitations of existing epigenetic studies. Interventions for obesity and diabetes can be carried out by supplementing corresponding nutrients in the perinatal period, but more explicit guidelines are needed. To detect the adverse effects of maternal micronutrient status, whether relevant epigenetic indicators can be utilized in obstetric examinations also needs more research. Thus, studies have great potential to understand the causes of the current diabetic epidemic and reduce the occurrence of diabetes worldwide.

## Author Contributions

YW drafted the manuscript. QZ and XX revised the manuscript. All authors contributed to the article and approved the submitted version.

## Funding

This work was supported by National Natural Science Foundation of China (Nos. 81870545, 81870579, 82170854, 81570715, and 81170736), Beijing Natural Science Foundation (7202163), Beijing Natural Science Foundation (7202163), Beijing Municipal Science and Technology Commission (Z201100005520011), National Key R&D Program of China (2017YFC1309603), National Key Research and Development Program of China (2016YFA0101002 and 2018YFC2001100), Scientific Activities Foundation for Selected Returned Overseas Professionals of Human Resources and Social Security Ministry, Beijing Dongcheng District Outstanding Talent Funding Project (2019DCT-M-05), Medical Epigenetics Research Center, Chinese Academy of Medical Sciences (2017PT31036 and 2018PT31021), the Non-profit Central Research Institute Fund of Chinese Academy of Medical Sciences (Nos. 2017PT32020 and 2018PT32001), and Chinese Academy of Medical Sciences Innovation Fund for Medical Sciences (CIFMS2017-I2M-1-008).

## Conflict of Interest

The authors declare that the research was conducted in the absence of any commercial or financial relationships that could be construed as a potential conflict of interest.

## Publisher's Note

All claims expressed in this article are solely those of the authors and do not necessarily represent those of their affiliated organizations, or those of the publisher, the editors and the reviewers. Any product that may be evaluated in this article, or claim that may be made by its manufacturer, is not guaranteed or endorsed by the publisher.
